# Obstetrics: The Science and Art

**Published:** 1849-07

**Authors:** 


### ARTICLE II.

*Obstetrics: the Science and Art.* By CHARLES D. MEIGS, M. D., Professor of Midwifery and the diseases of Women and Children in the Jefferson Medical College at Philadelphia; one of the Physicians to the Lying-in Department of the Pennsylvania Hospital; Vice-President of the Philadelphia College of Physicians; Member of the American Philosophical Society; of the American Medical Association, etc., etc. With one hundred and twenty-one illustrations. Philadelphia: Lea & Blanchard. 1849. pp. 685, 8vo. (From the Publishers---For sale by Griggs, Bross, & Co., Chicago.)

The announcement of the name of Dr. Meigs as the author of a work upon obstetrics, is a sufficient guarantee that it is something more than a mere compilation, as is the case with too many of our American Medical books recently published.

The long experience of Dr. Meigs, as a teacher and practioner in this department of medicine makes him fully capable of writing a useful and practical treatise of this kind. We were confidently expecting, therefore, to find in it many practical suggestions and useful hints for the obstetrician

After glancing over the work, we are happy in being able to say that we are not disappointed, and can, therefore, recommend it as being one of the very best treatises upon this subject and worthy of being placed in the library of every American physician. It deserves, and will most probably receive, in some future number of this journal, a more extended notice. H.

